# Supplementary material for: A Global Screen for Assembly State Changes of the Mitotic Proteome by SEC-SWATH-MS
Source: Cell Syst. 2020 Feb 26;10(2):133–155.e6. doi: 10.1016/j.cels.2020.01.001 (PMC7042714; doi:10.1016/j.cels.2020.01.001)

Q96QG7 | MTMR9_HUMAN | MTMR9 C8orf9 MTMR8
Monomer MW [kDa]: 63.462 Monomer expected elution fraction: 44

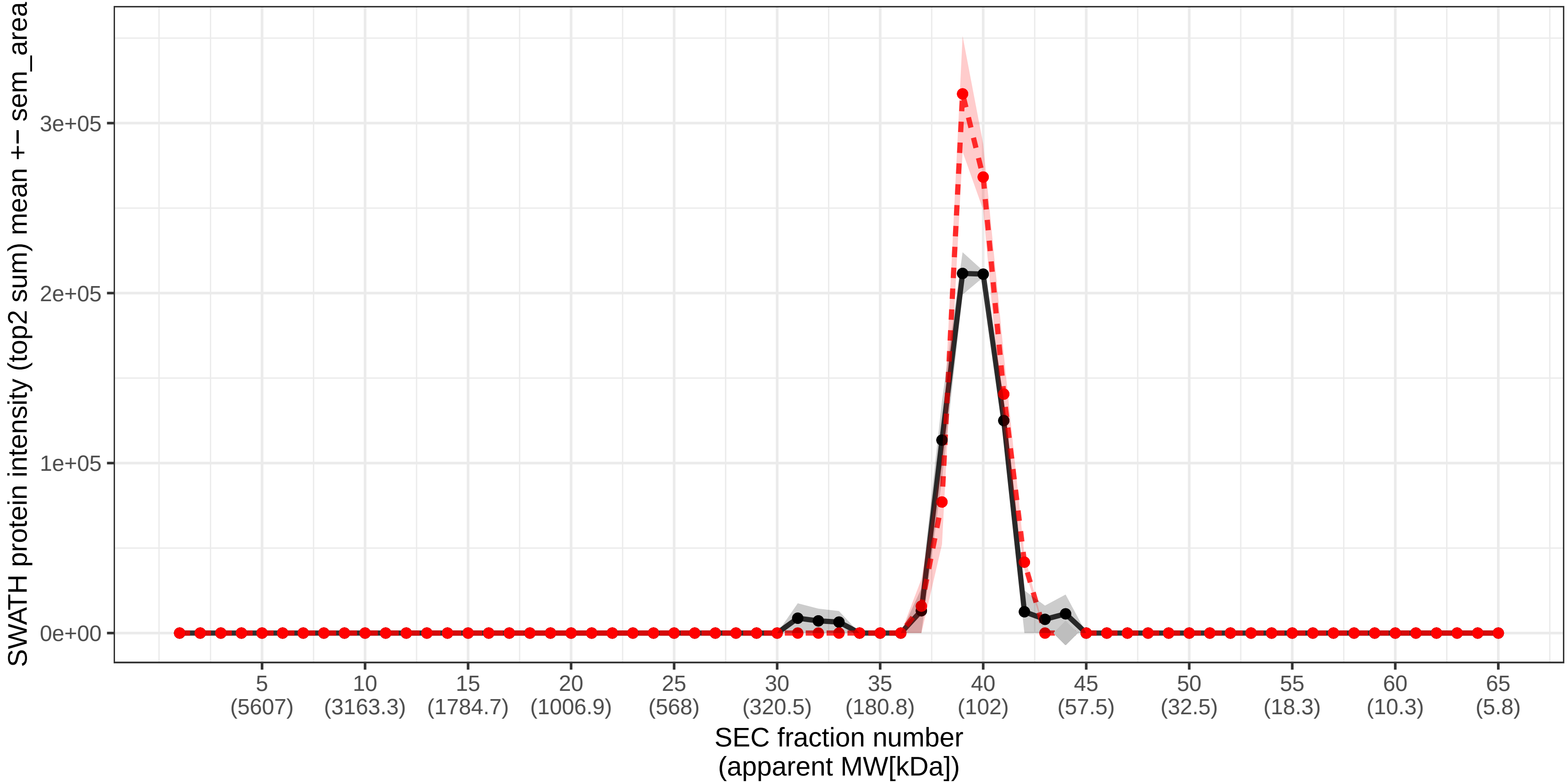

Supplement: Data S1. SEC-SWATH-MS Protein Chromatograms, Related to Figure 1 [file mmc6.zip › SECchrom_Q96QG7_MTMR9_HUMAN_MTMR9_C8orf9_MTMR8.pdf]
